# The independent effects of maternal obesity and gestational diabetes on the pregnancy outcomes

**DOI:** 10.1186/1472-6823-14-47

**Published:** 2014-06-13

**Authors:** Hayfaa A Wahabi, Amel A Fayed, Rasmieh A Alzeidan, Ahmed A Mandil

**Affiliations:** 1 Sheikh Bahmdan Chair of Evidence-Based Healthcare and Knowledge Translation, College of Medicine, King Saud University, P.O Box 102799, Riyadh 11685, Kingdom of Saudi Arabia; 2 King Saud Ben AbdulAziz University for Health Sciences, Riyadh, Kingdom of Saudi Arabia; 3 High Institute of Public Health Alexandria University, Alexandria, Egypt; 4 Department of Family and Community Medicine, King Saud University, Riyadh, Kingdom of Saudi Arabia

**Keywords:** Gestational diabetes, Maternal obesity, Cesarean section, Macrosomia

## Abstract

**Background:**

Obesity and gestational diabetes (GDM) in pregnancy are recognized risk factors for adverse outcomes, including cesarean section (CS), macrosomia and preeclampsia. The aim of this study was to investigate the independent effect of GDM and obesity on the adverse pregnancy outcomes at term.

**Methods:**

A retrospective cohort of postpartum women, in King Khalid University Hospital, were stratified according to body mass index (obese ≥30 kg/m^2^, non-obese <30 kg/m^2^) and the results of GDM screening into the following groups, women with no obesity and no GDM (reference group), women with no obesity but with GDM, women with obesity but no GDM and women with both GDM and obesity. Adverse pregnancy outcomes included high birth weight, macrosomia, CS delivery and preeclampsia. Multiple logistic regression used to examine independent associations of GDM and obesity with macrosomia and CS.

**Results:**

2701 women were included, 44% of them were obese and 15% had GDM. 63% of the women with GDM were obese. There was significant increase in the percentage of macrosomia, *P* < 0.001, high birth weight, *P* < 0.001, CS, *P* < 0.001 and preeclampsia, *P* < 0.001 in women with GDM and obesity compared to the reference group. Obesity increased the estimated risk of CS delivery, odds ratio (OR) 2.16, confidence intervals (CI) 1.74-2.67. The combination of GDM and obesity increased the risk of macrosomia OR 3.45, CI 2.05-5.81 and the risk of CS delivery OR 2.26, CI 1.65-3.11.

**Conclusion:**

Maternal obesity and GDM were independently associated with adverse pregnancy outcomes. The combination of both conditions further increase the risk.

## Background

Obesity is one of today’s major public health problems in both developed and developing countries. In 2005 there were around 400 million obese adults; this number is estimated to reach one billion by the year 2030 [1]. Reports from the Middle East [2] and Saudi Arabia [3] suggested similar burden of obesity epidemic. The prevalence of overweight and obesity is higher among women, with an estimated prevalence of 63% in the reproductive age group [3] and more than 50% among pregnant women [4]. Obesity in pregnancy is a recognized risk factor for many maternal and neonatal adverse outcomes including increased rate of cesarean section (CS), macrosomia, preeclampsia and gestational diabetes (GDM) [5-7].

The prevalence of GDM in Saudi Arabia is 12%-18% [8,9] which is one of the highest in the region and the world. Although there is an international agreement about the definition of GDM as “carbohydrate intolerance that begins or is first recognized during pregnancy” [10], there were many controversies about the adverse effects of GDM on the pregnancy outcomes and the need for screening and treatment of women who develop GDM [11]. However recent reports of randomized controlled trials and multicenter cohort studies, confirmed the need for control of hyperglycemia in women with GDM to improve the pregnancy outcomes [12] and that maternal hyperglycemia at levels even lower than those for diabetes mellitus (DM) are associated with adverse pregnancy outcomes in a linear relationship [13]. GDM is associated with many adverse pregnancy outcomes such as macrosomia and CS delivery [12,14], moreover and with recent developments in the research on fetal origin of adult disease, GDM has been linked to long term health effects on the mothers and their children including; increased risk of developing type 2 diabetes mellitus, maternal and childhood obesity and cardiovascular disease [15]. Few studies explored the relationship between GDM and maternal obesity and found an increased prevalence of GDM among obese women compared to those of normal weight [6], in addition obese women who develop GDM needed insulin to reach the target blood glucose level compared to normal weight women who were controlled by diet alone [16] and that the combination of obesity and GDM was associated with the worse outcomes compared to each condition alone [17,18]. A few studies investigated the independent effect of obesity and maternal hyperglycemia on the pregnancy outcome. Ricard et al., who investigated the independent effects of obesity and GDM on fetal weight, CS delivery and pregnancy- induced hypertension, found that obesity had greater independent effect on these adverse outcomes compared to GDM [18]. In their re- analysis of the HAPO study cohort, the research group reached a similar conclusion to that of Ricard et al.; however the greater impact of obesity was not consistent across all the studied adverse outcomes [17].

The aim of this study was to investigate the independent effect of GDM and obesity on the pregnancy outcomes at term in a Saudi population.

## Methods

This is a retrospective cohort study, designed to quantify the independent effects of maternal body mass index (BMI) and GDM on the pregnancy outcomes. Data were collected from all consented women who met the inclusion criteria and delivered in King Khalid University Hospital (KKUH), for the period of 12 months from the 1^st^ of July 2011 to 30^th^ of June 2012. The inclusion criteria for this study were:

1. Singleton pregnancy

2. Gestational age of ≥ 37 weeks at the time of delivery

3. Availability of documented records of maternal weight and height at the booking visit.

The exclusion criteria were:

1. Documented history of type 1 or type 2 diabetes mellitus prior to the index pregnancy.

2. Multiple- pregnancy.

3. Women who were not screened for gestational diabetes during the index pregnancy.

All women booked for antenatal care at KKUH are screened for pre-existing diabetes mellitus using fasting blood glucose (FBG) during their first antenatal visit. Values above 5.3 mmol/l indicate a full oral glucose tolerance test (OGTT). Further screening is carried out between 24–28 gestation weeks. Oral glucose (50 g) was administered, regardless of the time of the last meal. Venous plasma glucose was measured 1 h later. A value of 7.8 mmol/l (140 mg/dl) or more indicated the need for a full diagnostic OGTT. The diagnosis of GDM is based on the results of a 3-h, 100-g OGTT, interpreted according to the diagnostic criteria of Carpenter and Coustan [19]. Definitive diagnosis requires that two or more of the venous plasma glucose concentrations meet or exceed: fasting, 5.3 mmol/l (95 mg/dl), 1 h 10.0 mmol/l (180 mg/dl), 2 h 8.6 mmol/l (155 mg/dl) and 3 h 7.8 mmol/l (140 mg/dl). Once diagnosed, women with GDM follow a specific course of treatment including nutritional therapy and counseling together with antenatal fetal surveillance. Insulin therapy is introduced when nutritional therapy fails to maintain the FBG at 5.8 mmol/l (105 mg /100 ml) and/or the 2 h postprandial at 7.8 mmol/l (140 mg/dl).

Due to the proven effect of tobacco smoking, including environmental tobacco smoke (ETS) exposure, on the pregnancy outcomes, we collected data on maternal smoking status. We considered women to be exposed to ETS when the husband or one of the children smokes at home. Duration of exposure to ETS was not reported in this study as only 30% of the participants could recall the duration of exposure.

The BMI was calculated for each subject using the maternal weight and height recorded during the booking visit, according to the following equation; BMI = weight (kg)/height (m)^2^. Women were booked for their first antenatal visit during the first or the second trimester of pregnancy, subject to availability of appointments. To investigate the independent effect of maternal obesity and GDM on the pregnancy outcomes, women were divided into two groups based on the BMI, non-obese with BMI < 30 kg/m^2^ and obese with BMI ≥ 30 kg/m^2^. Further stratification of the study population into a total of four groups was based on the results of the GDM screening as follows; women with no GDM and who were not obese were considered the control group, second group was women who had GDM but were not obese, third group was women who were obese but did not have GDM and the fourth group were women with both obesity and GDM.

Data on the demographic and reproductive characteristics in addition to data on the outcomes of pregnancies were collected from the participants’ medical records, after delivery in the post-natal ward, using a pre-designed data collection sheet. Data on exposure to tobacco smoke were collected from the participants in the postnatal ward.

The maternal characteristics included in the study were; maternal age, parity, booking visit weight and height, smoking habits and exposure to ETS, the mode of delivery vaginal or cesarean section (CS), and the occurrence of GDM or pre-eclampsia (PE) defined as blood pressure ≥140/90 mm Hg after 20 weeks gestation and ≥ 0.3 g proteinuria/day [20], during the index pregnancy. The neonatal characteristics included; gestation age at delivery, APGAR scores at 5 minutes, birth weight, neonatal head circumference, neonatal length and admission to NICU.

### Primary outcomes

Fetal macrosomia (≥4 kg), birth weight, and CS delivery.

### Secondary outcomes

Secondary outcomes included; gestation age at delivery (≥37 weeks), the occurrence of PE during the index pregnancy, APGAR scores at 5 minutes, birth weight <2.5 kg, neonatal head circumference, neonatal length and admission to NICU.

### Statistical analysis

Data were analyzed using Statistical Package for the Social Sciences (SPSS), Version 17(SPSS Inc., Chicago, IL, USA). We compared means using Analysis Of Variance (ANOVA). After assessing normality of distribution of the variables, either *Pearson's chi-square* test or Fisher exact test was used for categorical variables as indicated. *P*-value of less than 0.05 considered statistically significant.

Stepwise multivariate logistic regression models were used to explore the independent associations between the four groups of combination of maternal obesity and GDM with adverse pregnancy outcomes, considering women who were not obese and did not have GDM as the reference group. Adjusted odd ratios (OR) were calculated. The following variables were included as confounders for the final model for macrosomia and CS delivery; maternal age, parity, ETS exposure and gestation age (≥37 weeks).

### Ethical approval

Ethical approval number 11/2862/IRB, was obtained for this study from institutional review board of the Collage of Medicine King Saud University before the commencement of the study.

## Result

### Maternal characteristics and pregnancy outcomes

Of the 3200 women who delivered during the study period, 2701 met the inclusion criteria and consented to the study. The mean BMI of the study population at booking was 30.25 ± 14.99 kg/m^2^ and 44% of the participants were obese. The prevalence of GDM was 15%, and 63% of the women who were diagnosed with GDM were obese (Table 1). Of the study population 869 (32.2%) were exposed to ETS during the index pregnancy. The mean gestation age at booking was 21.8 ± 8.8 weeks; the mean maternal age was 29.38 ± 6.21 years. More than 50% of the participants were university graduates or above, however 76.1% of the women in this study were housewives (Table 1). The characteristics of the neonates in this study are shown in Table 1.

**Table 1:** Maternal and neonatal characteristics of the studied population

**Maternal characteristics**	
Age (year)	29.38 ± 6.21
Gravidity	3.50 ± 2.65
Parity	2.99 ± 2.18
Gestation age at booking (week)	21.8 ± 8.8
Education	
Illiterate	58 (2.1)
School	1281 (47.5)
University and above	1362 (50.4)
Work	
Housewife	2055 (76.1)
Student	311 (11.5)
Employee	335 (12.4)
BMI (kg/m^2^)	30.25 ± 14.99
Obesity/GDM groups	
No GDM No Obesity	1361 (50.4)
GDM No Obesity	155 (5.7)
Obesity No GDM	925 (34.2)
GDM and Obesity	260 (9.6)
ETS exposure	
Not exposed	1832 (67.8)
Exposed	869 (32.2)
**Neonatal characteristics**	
Gestational age at delivery (week)	39.11 ± 1.17
Birth weight(kg)	3.19 ± 0.46
Head circumference (cm)	34.44 ± 10.39
Length (cm)	49.74 ± 2.56
Gender (male)	1429 (52.9)

Data expressed as n (%) or mean ± Standard Deviation.

GDM = gestational diabetes mellitus, BMI = body mass index, ETS = environmental tobacco smoking.

Univariate analysis showed statistically significant association between maternal obesity and GDM with adverse pregnancy outcomes (Table 2). There was significantly increased percentage of CS delivery, *P* <0.001, macrosomia, *P* <0.001, PE, *P* <0.001, increased birth weight *P* <0.001, and length of the newborn, *P* <0.01, for GDM or obesity alone compared to the reference group. The combination of GDM and obesity showed considerably higher percentages of adverse outcomes compared to either GDM or obesity alone (Table 2). There was a noticeable trend of higher percentages of adverse pregnancy outcomes with obesity alone compared to GDM alone (Table 2).

**Table 2:** The effects of Obesity/GDM on the pregnancy outcomes of terms infants

	**Obesity/GDM groups**				
**Pregnancy outcome**	**No Obesity No GDM**	**GDM No Obesity**	**Obesity No GDM**	**Obesity**	
				**GDM**	**p-value**
**Caesarean section**	210 (15.4)	27 (17.4)	263 (28.4)	83 (31.9)	<0.001
**Pre-eclampsia**	7 (0.5)	3 (1.9)	14 (1.5)	9 (3.5)	<0.001
**Gestational age (weeks)**	39.15 ± 1.16	38.89 ± 1.00	39.17 ± 1.24	38.86 ± 1.08	<0.001
**APGAR score at 5 min**	8.91 ± 0.69	8.93 ± 0.57	8.93 ± 0.56	8.92 ± 0.69	0.90
**Birth weight (kg)**	3.13 ± 0.44	3.17 ± 0.41	3.22 ± 0.45	3.33 ± 0.54	<0.001
**Macrosomia ≥4 kg**	40 (2.9)	4 (2.6)	47(5.1)	32 (12.3)	<0.001
**Low birth weight <2500 g**	85 (6.2)	8 (5.2)	38 (4.1)	10 (3.8)	0.01
**Newborn length (cm)**	49.65 ± 2.21	49.29 ± 4.50	49.87 ± 2.70	50.0 ± 2.35	0.01
**Newborn head circumference (cm)**	34.23 ± 8.87	33.72 ± 3.02	34.89 ± 14.05	34.37 ± 1.46	0.384
**NICU admission**	61 (4.5)	9 (5.8)	48 (5.2)	15 (5.8)	0.79

NICU = Neonatal Intensive care.

Data is expressed either as number (%) or mean **±** standard deviation.

The combination of GDM and obesity increased the odds of delivering a macrosomic baby by nearly fourfold, OR 3.45, Confidence Intervals (CI) (2.05-5.81) (Table 3). Obesity alone increased the estimated risk of delivering a macrosomic baby to 1.46, however this risk was not statistically significant, CI 0.94-2.27, *P* = 0.092 (Table 3).

**Table 3:** Adjusted ORs for macrosomia and cesarean section delivery in obese and non-obese women with and without gestational diabetes

**Outcome**	**OR**	**CI**	**p-value**
**Macrosomia**			
No Obesity No GDM	1.00		
No Obesity GDM	0.74	0.26-2.13	0.584
Obesity No GDM	1.46	0.94-2.27	0.092
Obesity GDM	3.45	2.05-5.81	<0.001
**Cesarean section delivery**			
No Obesity No GDM	1.00		
No Obesity GDM	0.97	0.62-1.53	0.91
Obesity No GDM	2.16	1.74-2.67	<0.001
Obesity GDM	2.26	1.65-3.11	<0.001

GDM = Gestational Diabetes Mellitus, BMI = Body Mass Index, OR = Odds Ratio.

Reference group: non diabetic non-obese women (BMI <30 kg/m^2^.

Adjusted for maternal age, parity, gestation age and exposure to environmental tobacco smoke.

The estimated risk of having a CS delivery was increased by more than twofold in obese mothers compared to those who were non-obese and did not develop GDM, OR 2.16, CI 1.74-2.67, *P* < 0.001 (Table 3). The combination of GDM and obesity had similar risk of CS delivery to that of obesity alone, OR 2.26, CI 1.65-3.11, *P* < 0.001 (Table 3).

## Discussion

The results of this study showed that GDM and maternal obesity were independently associated with adverse pregnancy outcomes. The findings confirmed that the combination of both GDM and obesity had greater impact on macrosomia and CS delivery than either obesity or GDM alone. In addition there was a noticeable trend of increment in maternal and neonatal adverse outcomes in mothers with obesity alone compared to those with GDM alone. The greater impact of maternal obesity on the adverse pregnancy outcomes has been reported by other investigators [6,21].

While fetal weight is influenced by the parents’ weight and height through a genetic link, maternal weight has an additional influence by modulating the intrauterine environment [22]. Maternal obesity is associated with hyperinsulinemia, hyperlipidemia and increased inflammatory markers levels [23]. The level of circulating maternal lipids near delivery, rather than the maternal glucose level, was confirmed to be a strong predictor of fetal size [18,24,25]. This explains our findings of the greater influence of maternal obesity, both alone and in combination with GDM, on macrosomia, compared to the influence of GDM alone.

The high prevalence of 15% for GDM in this study is consistent with previous reports from Saudi Arabia [8,9] and is not surprising considering the high prevalence of obesity among the obstetric population reported in this study. Our results pointed to modest effect of GDM on macrosomia compared to maternal obesity, these findings are consistent with the findings of Ricard et al. [18]. The pathophysiology of macrosomia in women with GDM is based on Pedersen hypothesis [26] of maternal hyperglycemia leading to fetal hyperinsulinemia and increased utilization of glucose and hence increased fetal adipose tissue. The hypothesis was further supported by the finding of high insulin levels in the cord blood of babies born to diabetic mothers [27]; however good control of blood glucose did not completely prevent the increase in macrosomia noticed in women with diabetes [28], which suggests a role for other mediators that might share a common pathway with obesity .

In this study we found an independent association between the frequency of CS delivery and maternal obesity with twofold increase in the estimated risk for CS delivery for obese women compared to the reference group. It is worth mentioning that GDM has not imposed extra risk for CS delivery over that estimated for maternal obesity. A recently published systematic review reported an estimated two to threefold increase in the rate of CS for obese women compared to those with normal weight [29]. Similar findings were reported by Dietz et al. even after adjustment for co-morbidity, such as GDM [30]. This finding is relevant considering the increasing rate of CS delivery [31] and obesity in many of the middle and high income countries [4,5], taking into account the significant morbidities in obese women who deliver by CS such as wound infection, endometritis, urinary tract infection and prolonged postpartum hospitalization [32,33].

The mechanism behind the increased frequency of CS in obese mother has not been fully explored, however cephalopelvic disproportion due to macrosomia and narrow maternal pelvis due to increased soft tissue mass [34], slow labour progression [35] and increased frequencies of emergencies [36] were all proposed as explanations for the association of maternal obesity and CS delivery.

The prevalence of maternal obesity in this study was 44% which is higher than that reported for Saudi obstetrics population but similar to at risk population of over weight and obese mothers from the same report [4]. Marked increase in the prevalence of maternal obesity in the last decade was reported from other developed countries and the region [6,37]. Our findings indicated that most of the study population received formal education and more than 50% of the participants were university graduates; however more than 75% of the participants did not work for pay which might indicate the predominance of sedentary life style in this cohort, considering the high standard of living in Saudi Arabia (Gross national income per capita is $30,160) [38] and the availability of household help for domestic work.

Few reports investigated interventions to improve the outcomes in pregnancies complicated by maternal obesity. Recently published systematic reviews have shown that dietary interventions during pregnancy are effective in the prevention of maternal obesity and its adverse effects on the mother and the newborn [39,40].

Based on the results of this study and previous similar reports [18,25], we believe it is prudent for maternity health services worldwide to implement evidence based intervention for reducing and preventing obesity in pregnancy and its adverse effects on the mother and the fetus. Greater efforts should be directed towards investigating effective interventions for reducing maternal weight before pregnancy, such as bariatric surgery. It is important that women in the reproductive age group are aware of the adverse consequence of obesity on the pregnancy outcomes which will motivate better utilization of effective interventions.

We are aware of the limitations of this study including the retrospective nature of the investigation and the lack of data on pre-pregnancy maternal weight due to the routine late booking for antenatal care in the hospital, considering that maternal adiposity, which was the proposed influence of adverse pregnancy outcomes in this study, correlates better with maternal pre-pregnancy and first trimester weight rather than maternal weight in late pregnancy [41]. However other measurements of maternal obesity such as gestational weight gain, central obesity and modified categories of BMI to define obesity during pregnancy, were found to be associated with macrosomia and increased risk of CS [17,42,43]. This limitation of the study might have attenuated the effects of obesity in outcomes other than macrosomia and CS because of the variation in maternal weight gain during pregnancy; however it would not produce false positive results. The prevalence of obesity in this study is higher than previously reported, which might be due to the late booking of the women for antenatal care during the second trimester, this might limit the generalization of the results to the whole pregnant population of the Kingdom.

## Conclusion

Maternal obesity and GDM were independently associated with adverse pregnancy outcomes. The combination of both conditions further increase the risk of adverse pregnancy outcomes at term.

## Competing interest

The authors declare that they have no conflict of interest.

## Authors’ contributions

HW conceived the idea of the study, was responsible for writing the final study manuscript. AF was responsible for the statistical analysis; she participated in writing the draft of the manuscript. RZ participated in writing the draft of manuscript and AM reviewed the article. All authors reviewed and approved the final manuscript.

## Pre-publication history

The pre-publication history for this paper can be accessed here:

http://www.biomedcentral.com/1472-6823/14/47/prepub
